# Response Surface Optimization of Bioethanol Production from Sugarcane Molasses by* Pichia veronae* Strain HSC-22

**DOI:** 10.1155/2015/905792

**Published:** 2015-12-08

**Authors:** Hamed I. Hamouda, Hussein N. Nassar, Hekmat R. Madian, Salem S. Abu Amr, Nour Sh. El-Gendy

**Affiliations:** ^1^Egyptian Petroleum Research Institute, P.O. Box 11727, Nasr City, Cairo, Egypt; ^2^Malaysian Institute of Chemical and Bioengineering Technology, Universiti Kuala Lumpur, Melaka, Malaysia

## Abstract

*Pichia veronae* strain HSC-22 (accession number KP012558) showed a good tolerance to relatively high temperature, ethanol and sugar concentrations. Response surface optimization based on central composite design of experiments predicted the optimal values of the influencing parameters that affect the production of bioethanol from sugarcane molasses to be as follows: initial pH 5, 25% (w : v) initial molasses concentration, 35°C, 116 rpm, and 60 h. Under these optimum operating conditions the maximum bioethanol production on a batch fermenter scale was recorded as 32.32 g/L with 44% bioethanol yield.

## 1. Introduction

Recently, the worldwide application of biofuels as alternative or complementary for petrofuels has grown. This is due to the limitation of oil reserves, fluctuation of oil price, the increased concern about the global warming and climate change caused by the increment of the greenhouse gas emissions, and the awareness to promote rural economics [1]. Ethanol, in spite of its lower heating value than gasoline, it has become as one of the most important renewable fuels in the worldwide markets, due its economic and environmental benefits [2].* Saccharomyces cerevisiae* has been the most commonly used microorganism for the ethanol production by the alcoholic fermentation of different feedstock rich in sugars [1, 3, 4]. From the economic view point, molasses as an agroindustrial waste is widely used as a raw material for the production of ethanol [1, 3–11]. To our knowledge, there is no report published on the bioethanol fermentation from molasses by* Pichia veronae*.

A number of factors like high temperature, low ethanol, and sugar tolerance of the yeast limit the industrial production of ethanol at low production costs. The use of concentrated sugar substrate is one of the ways to obtain high ethanol yield during fermentation. However, high substrate concentrations are inhibitory to fermentation due to the osmotic stress. Also, it is known that the pH of the fermentation medium significantly affects the process [10, 12]. The usage of multivariant techniques for the optimization of processes has increased since the past few years [4, 9–11].

The aim of this study is to optimize the production of ethanol by Egyptian yeast strain* Pichia veronae* HSC-22 (accession number KP012558) on the batch flasks scale. Response surface methodology RSM based on central composite design CCD of experiments was used in this study, to overcome the limitation of one-at-a-time-parameter optimization. The production of bioethanol under the obtained optimum operating conditions was also investigated on a fermenter scale.

## 2. Materials and Methods

### 2.1. Feedstock

Sugarcane molasses was purchased from Sugars and Integrated Industries Egyptian Distillation Plants in Hawamdia City, Giza, Egypt, and stored at 4°C until use.

### 2.2. Media

Wickerham WH medium prepared according to Wickerham [13] was used for maintenance and inoculum preparation.

Medium for fermentation experiments was prepared as follows: 2 g KH_2_PO_4_, 10 g (NH_4_)_2_SO_4_, 1 g MgSO_4_·7H_2_O, and 2 g yeast extract were dissolved in 1 L distilled water and molasses and pH values were then adjusted according to the experimental conditions (Table 1), before sterilization, at 121°C for 20 min to avoid contamination.

### 2.3. Microorganism and Inoculum Preparation

The yeast* Pichia veronae* strain HSC-22 (accession number KP012558) used in this study was obtained from Petroleum Biotechnology Lab, Egyptian Petroleum Research Institute EPRI. Active cultures for fermentation experiments were prepared by growing HSC-22 in WH medium for 48 h at 30°C in shaking incubator 150 rpm. Harvested cells were washed twice with sterile saline (8.5 g NaCl per 1 L distilled water) and then resuspended in sterile saline to be used as a fresh and pure stock for inoculation.

### 2.4. Analytical Methods

The types and concentration of sugars in molasses were determined using high performance liquid chromatography HPLC (1200 Series Agilent HPLC, USA) equipped with a refractive index RI detector (model Agilent 1260 infinity, USA) and Spherisorb Amino (NH_2_) Cartridge column (pore size 80 Å, inner diameter 4.6 mm, length 250 mm, and particle size 5 *μ*m, Waters, Ireland). The mobile phase was acetonitrile : water (80 : 20 v/v), flow rate was 1.5 mL/min, and injection volume was 10 *μ*L and the column temperature was 35°C. Ethanol concentration (g/L) was measured by gas chromatography (model 6890 (G1530A), Agilent, USA), equipped with flame ionization detector and nominal capillary column (HP-5, 5% phenyl- 95% methylsiloxane 30 m × 250 *μ*m ID, 5.00 *μ*m film, USA). Nitrogen was the carrier gas; flow rate was 25 mL/min. Oven and detector temperature was 300°C, and the bioethanol yield was calculated according to the following equation:

(1) Bioethanol  yield %=Produced  bioethanol  concentration  g/LAmount  of  total  sugars  in  the  substrate  g/L×100.

The fermentation efficiency was calculated according to El-Refai et al. [6]:

(2) $$\begin{array}{c} \frac{Actual\;\;ethanol\;\;content}{Theoretical\;\;ethanol\;\;content}\times 100, \end{array}$$

where the theoretical ethanol content = total fermentable sugar × 0.64 [14].

All of the aforementioned analyses were done in Central Analytical Lab, Egyptian Petroleum Research Institute. All other chemical characterizations of molasses were done in Agricultural Research Center, Giza, Egypt. Estimation of total reducing sugars in the collected molasses samples was carried out by 3,5-dinitrosalicylic acid (DNS) [15]. All experiments were carried out in triplicate, and the listed results are the average.

### 2.5. Fermentation Experiments

Batch fermentations were done in 100 mL Erlenmeyer flasks fitted with rubber stoppers, containing 50 mL of culture media with different molasses concentrations (wt.%) and pH values, adjusted according to the required experimental conditions (Table 1), and were inoculated with 10% (v/v) yeast suspension (≈10^5^ cells/mL). Incubation was performed in shaking incubator, set at different temperatures and shaking speeds according to the required experimental conditions. Samples for analyses were taken at the beginning and end of fermentation at different prescribed incubation periods.

### 2.6. Experimental Design

Response surface methodology (RSM) was used to optimize bioethanol production process from sugarcane molasses (SCM) and investigate the influence of different fermentation process variables on the bioethanol yield. The central composite design CCD was applied to study process variables. The experimental runs were carried out according to a 2^5^ full factorial design for the five identified design independent variables, namely, initial pH (*A*), molasses concentration wt.% (*B*), incubation temperature °C (*C*), mixing rate rpm (*D*), and incubation period h (*E*), with low (−1) and high (+1) levels. The total number of experiments (runs) was given by the simple formula  [50 = 2^*k*^ + 2*k* + 8], where *k* is the number of independent variables (*k* = 5); this includes the following: 32 factorial points from 42 full factorial CCD were augmented with 8 replicates at the center point to assess the pure error. Response selected was bioethanol yield. The levels were selected based on preliminary study results. The design factors (variables) with low −1 and high +1 levels are, namely, *A* [4 and 6], *B* [15 and 25 wt%], *C* [25 and 35°C], *D* [50 and 150 rpm], and *E* [24 and 72 h]. The central values (zero levels) chosen for experimental design were as follows: pH 5, 20%, 30°C, 100 rpm, and 48 h for *A*, *B*, *C*, *D*, and *E*, respectively (Table 1).

### 2.7. Statistical Analysis

Once the experiments were preformed, the next step was to perform a response surface experiment to produce a prediction model to determine curvature, detect interactions among the design factors (independent variables), and optimize the process, that is, determine the local optimum independent variables with maximum yield of bioethanol. The model used in this study to estimate the response surface is the quadratic polynomial represented by the following equation:

(3) $$\begin{array}{c} Y=\beta_{o}+\overset{5}{\underset{i=1}{\sum }}\beta_{i}x_{i}+\overset{4}{\underset{i=1}{\sum }}\;\overset{5}{\underset{j=i+1}{\sum }}\beta_{ij}x_{i}x_{j}+\overset{5}{\underset{i=1}{\sum }}\beta_{ii}x_{i}^{2}, \end{array}$$

where *Y* is the bioethanol yield (g/L), *β*_*o*_ is the value of the fixed response at the center point of the design, and *β*_*i*_, *β*_*ij*_, and *β*_*ii*_ are the linear, interactive, and quadratic coefficients, respectively. *x*_*i*_ and *x*_*j*_ are the independent variables (factors) under study.

The statistical software Design Expert 6.0.7. (Stat-Ease Inc., Minneapolis, USA) was used for design of experiments, regression, and graphical analysis of the data obtained and for statistical analysis of the model to evaluate the analysis of variance (ANOVA) and it was used also for the optimization of the bioethanol fermentation process.

### 2.8. Batch Fermentation under Optimum Conditions

A batch fermentation of SCM was performed under the selected optimum conditions, in a self-sterilizer 10 L bioreactor (Biotron Liflus SL, Korean Republic) with a working capacity of 5 L; after the sterilization step, the broth was cooled and then inoculated with 10% (v/v) yeast suspension (≈10^5^ cells/mL). The batch fermentation was conducted for 72 h, and the produced ethanol and residual sugars' concentrations were determined, during the prescribed time intervals.

## 3. Results and Discussion

### 3.1. Chemical Composition of Molasses

Sugarcane molasses was a dark viscous fluid with pH value of 5 and very rich in nutrients required by most microorganisms. Carbon, nitrogen, phosphors, sodium, and potassium contents were 64, 6, 0.5, 0.9, and 5.5 (wt.%), respectively. Nonnitrogenous compounds (e.g., citric acid, oxalic acid) represented 2–8% (wt.%). Molasses had no furfural which is toxic to most of fermenting microorganisms. The ashes (11% wt.%) constitute a source of mineral elements. Molasses was found to be rich in calcium ≈1.7% and contained significant quantities of trace minerals: copper (5.4 ppm), zinc (8.8 ppm), manganese (11.6 ppm), iron (190.6 ppm), and magnesium (3379 ppm). It had total sugars of 292.82 g/L, where sucrose recoded the largest percentage of 70.97%, followed by glucose 16.54%, fructose 9.67%, xylose 2.10%, and maltose 0.72%. The SCM contained total reducing sugars TRS (69.6 g/L). The SCM was rich in fermentable sugars ≈55% (wt%) and the nonfermentable sugars recorded ≈5% (wt%).

Most of the chemical parameters determined in this study were in close agreement with those reported by Chen and Chou [16], who found that molasses contains 45–55% total sugars, 20–25% reducing sugars, 10–16% ash, 0.4–0.8% calcium, 0.1–0.4% sodium, 1.5–5% potassium, and pH 5–5.5. These results were in agreement with those reported by Nakata et al. [17], where the main compound in cane molasses is sucrose, while glucose and fructose are found in lower concentrations.

### 3.2. Regression Model and Its Validation

The main concern in this study is the actual amount of produced bioethanol, that is, the actual yield of bioethanol relative to the amount of total sugars in the initial substrate (molasses) concentration.

The complete design matrix with experimental and predicted values of the produced bioethanol yield (%) is presented in Table 2. Based on CCD and experimental data, the following second-order quadratic model equation describing the influence of different considered variables on process yield was obtained:

(4) Y=35.4+0.509A+1.63B+1.45C+1.84D+4.28E−12.3A2+1.86B2+0.855C2+0.48D2−9.98E2−0.244AB−0.676AC+0.394AD−0.0294AE+0.166BC−1.02BD+0.618BE−0.308CD+0.392CE+1.25DE,

where *Y* is the bioethanol yield % and positive sign in front of the terms indicates synergetic effect, whereas negative sign indicates antagonistic effect.

**Figure 1:**
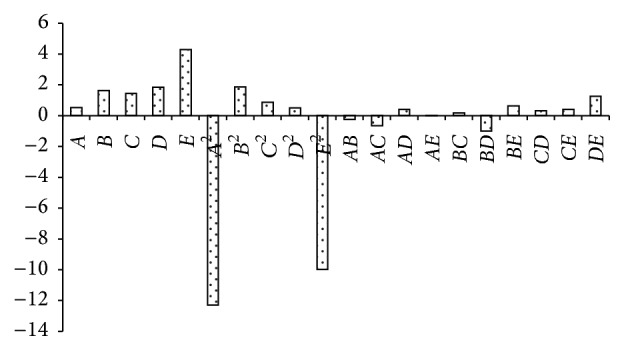
Pareto chart showing the effect of different independent variables on bioethanol yield.

Pareto charts, which are very useful in design of experiments, were used in this work, to make it much easier to visualize the main and interaction effects of all factors to the response variable, that is, bioethanol yield (Figure 1). The model identified that within the studied range of experiments the incubation period has the highest positive impact on the fermentation process followed by the mixing rate, initial molasses concentration, and incubation temperature, in a decreasing order. While the initial pH has a slight positive impact on the bioethanol yield (%), its quadratic effect has the highest negative impact on the fermentation process, followed by the negative quadratic effect of incubation period. While the quadratic effects of the initial molasses concentration, incubation temperature, and mixing rate have a positive impact on the fermentation process in a decreasing order, the positive interactive effect of the studied parameters can be ranked in the following decreasing order: mixing rate and incubation period > initial molasses concentration and incubation period > initial pH and mixing rate ≈ incubation period and temperature > initial molasses concentration and incubation temperature. But the negative interactive effect of the studied parameters can be ranked in the following decreasing order: initial molasses concentration and mixing rate > initial pH and incubation temperature > incubation temperature and mixing rate > initial pH and molasses concentration > initial pH and incubation period.

The validity of the fitted model was evaluated and the statistical significance was controlled by *F*-test. The analysis of variance (ANOVA) for the response surface full quadratic model is given in Table 3. It can be indicated that the model (4) is very highly statistically significant at 95% confidence level, with *F*-value of 29.1 and very low probability *P* value of < 0.0001; that is, there is less than 0.01% chance that this error is caused by noise. The values of the determination coefficients, *R* ^2^ and *R*_adj_^2^, which measure the model fitting reliability, were calculated and found to be 0.953 and 0.920, respectively. This suggests that approximately 95.3% of the variance is attributed to the variables, which indicated the high significance of the model, where only 4.7% of the total variations cannot be explained by the model (4), which ensures the good adjustment of the above predicted model to the experimental data. Confirmation of the adequacy of the regression model was reflected also by the good agreement between the experimental and the predicted values of the response variables as shown in Table 2, where the experimental bioethanol yield ranged from 8.20 to 41.4% and the corresponding predicted values were 9.26 and 39.1%, respectively. The “Adeq Precision” measures the signal to noise ratio. A ratio greater than 4 is desirable. The ratio of 17.1 for model (4) indicated the adequate signal. This model is reliable and can be used to navigate the design space. The standard deviation SD and the coefficient of variance were low, recording 2.87 and 12.8 for model equation (4), respectively.

**Figure 2:**
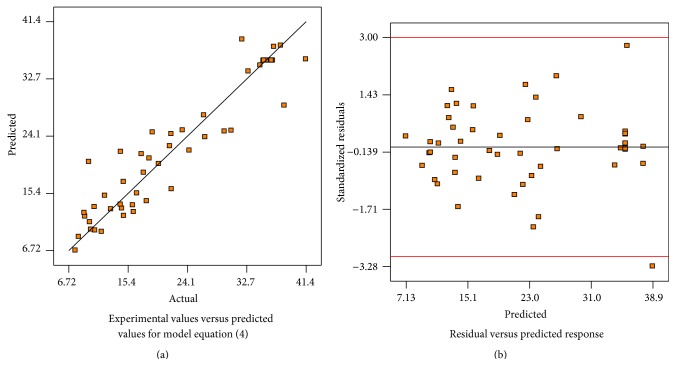
Validity of model equation (4).

The performance of the model can be observed by the plots of the predicted versus experimental results of bioethanol yield (Figure 2(a)), which showed high correlation coefficients (*R* ^2^ = 0.94), indicating that the predicted and experimental values were in reasonable agreement. This means that the data fit well with the models and give a convincingly good estimate of response for the system in the studied experimental range.  Figure 2(b) presents a plot of the residual distribution, defined as the difference between calculated and observed values of the response variable studied, versus predicted response. The quality of the fit is good because the residual distribution does not follow a trend with respect to the predicted values of response variable, which indicate that the quadratic model adequately represents the bioethanol % yield over the studied experimental range.

**Figure 3:**
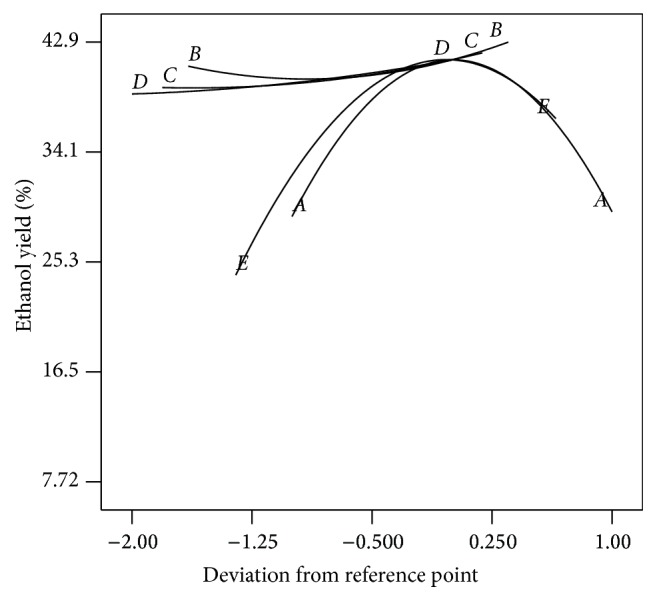
Perturbation plot.

The perturbation plot (Figure 3) shows the comparative effects of all the studied independent variables on the bioethanol yield %. The curvatures of the five studied factors from the center point confirm the statistical data obtained from analysis of variance (ANOVA, Table 3), that is, the significance of each parameter (coefficient). It is obvious from the sharp curvature of the independent variables initial pH (*A*) and incubation period (*E*, h) that the bioethanol yield increased with the increment of initial pH and incubation period until reaching the central point around pH 5 and 48 h and then the yield sharply decreased with higher pH but the longer incubation period caused a relative decrease in the bioethanol yield. The comparatively low curvature of initial molasses concentration (*B*, % w : v), incubation temperature (*C*, C°), and mixing rate (*D*, rpm) showed less sensitivity of bioethanol yield towards the changes in these three factors and the increase of bioethanol yield with the increment of these three parameters. The curvatures also confirm the data illustrated in Pareto chart (Figure 1). This was also confirmed by the analysis of variance (ANOVA) of the regression model, where the statistical significance of the main and interacting effects of different studied parameters on the bioethanol yield at 95% confidence level was studied and illustrated in Table 3. The significance of each coefficient was determined by *F*-values and *P* values. The larger the magnitude of the *F*-value, the smaller the *P*-values and the more significant the corresponding coefficient. This implies that the main effect of mixing rate, incubation temperature, and initial molasses concentration has statistically positive impact on bioethanol yield (*P* = 0.0008, 0.006, and 0.002, resp.), while the quadratic effects of initial pH and incubation period have a very highly negative statistically significant effect on bioethanol yield (*P* < 0.0001), that is, decrease in bioethanol yield with increment of these parameters. But the interactive effect of the mixing rate and incubation period has a statistically possible positive impact on the bioethanol yield (*P* = 0.0195), while the interactive effect of the initial molasses concentration and mixing rate has a statistically possible negative impact on the bioethanol yield (*P* = 0.0534).

### 3.3. Optimization of the Fermentation Process

Three-dimensional response surfaces were plotted on the basis of the predicted model equation to investigate the interaction among the variables and to determine the optimum condition of each factor for maximum bioethanol yield %.

**Figure 4:**
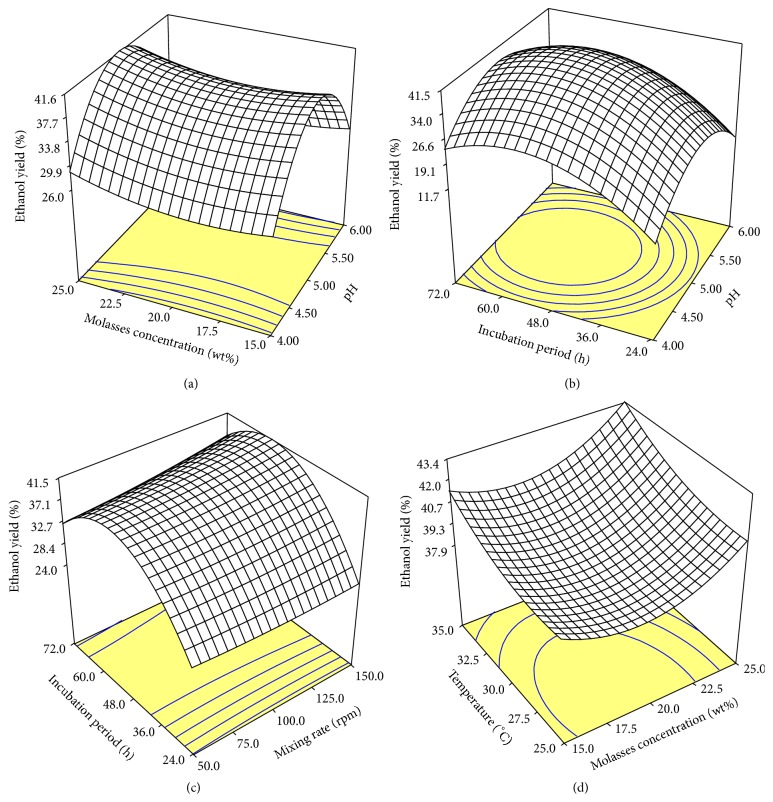
RSM and contour plots.

It is obvious from the RSM and contour plots of the interactive effect of the initial molasses concentration and pH (Figure 4(a)) that with the increase of initial pH the bioethanol yield increased reaching its maximum within pH 5 but decreased again with further increase of initial pH, while the bioethanol yield increased with the increment of the initial molasses concentration, recording maximum bioethanol yield at pH 5 and 25% initial molasses concentration. The elliptical shape (Figure 4(b)) showed the negative interactive effect of initial pH and incubation period on the bioethanol yield, where the bioethanol yield increased with the increment of the incubation period and initial pH and recorded its maximum of ≈42% at pH 5 within 48–60 h incubation period but decreased with further increment in initial pH and incubation period. But the RSM and contour plots (Figure 4(c)) showed the positive interactive effect of the mixing rate and incubation period on the bioethanol yield, where the bioethanol yield increased with the increment of mixing rate and incubation period recording its maximum within 48–60 h but decreased with longer incubation period. The plots showed also that the bioethanol yield was not highly increased at higher mixing rate (≥120 rpm).  Figure 4(d) shows the positive interactive effect of molasses concentration and incubation temperature, where the bioethanol yield increased with the increment of those two factors recording its maximum of ≈44% at 25% initial molasses concentration at 35°C.

Maiorella et al. [18], Cazetta et al. [19], and Shafaghat et al. [11] reported the pH of fermentation medium as an important parameter affecting the microbial growth and product formation. Misono and Yamaguchi [5] reported that the optimum pH for the alcoholic fermentation of molasses was pH 5 and decreased with the increase of pH to pH 6. Yadav et al. [9] reported molasses fermentation by* S. cerevisiae* HAU-1, where the increase in pH from 4 to 5 increased the alcohol productivity and concentration, while the optimum pH range was within pH 4.5–5. Morimura et al. [8] reported a yeast strain K211 that showed highest cell viability and ethanol productivity in a molasses medium containing 25% (w : v) at 35°C. Cazetta et al. [19] reported maximum bioethanol fermentation of molasses* by Zymomonas mobilis* at 35°C and higher temperature has negative impact on fermentation process. Shafaghat et al. [11] reported maximum bioethanol fermentation of molasses by* Saccharomyces cerevisiae* PTCC24860 at pH 5.3 and incubation time of 24 h, where bioethanol production decreased at longer incubation period, due to sugar depletion, ethanol oxidation, and organic acid formation. Dombek and Ingram [20] reported that the accumulation of ethanol in fermentation broth causes deactivation of alcohol producing enzymes.

The optimization process was carried out to determine the optimum values of the studied five parameters affecting the fermentation process of sugarcane molasses by* Pichia veronae* strain HSC-22, to maximize the bioethanol production (g/L) and bioethanol yield %. This was done using Design Expert 6.0.7 software (Stat-Ease Inc., Minneapolis, USA). According to the software optimization step, the desired goal for each fermentation parameter (*A* initial pH, *B* initial molasses concentration wt%, *C* incubation temperature °C, *D* mixing rate rpm, and *E* incubation period h) was defined within the studied levels range to achieve the highest performance. The program combines the individual desirability into a single number and then searches to optimize this function based on the response goal. Accordingly, the optimum conditions giving the maximum calculated bioethanol production of 30.7 g/L with bioethanol yield of 42% were as follows: pH 5, 25% initial molasses concentration, 35°C, 116 rpm, and 60 h, with desirability function value of 1. The experimental result of these conditions was found to be 32 g/L with bioethanol yield of 43.57%. That indicates the process optimization based on CCD of experiments was capable and reliable to optimize the bioethanol fermentation process of SCM by* Pichia veronae* strain HSC-22.

### 3.4. Batch Fermentation under Optimum Conditions

**Figure 5:**
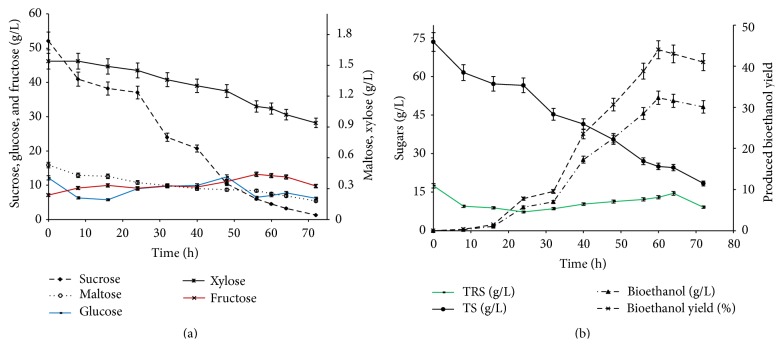
Time profile of sugars consumption and bioethanol production.

After optimizing the various parameters, pH, molasses concentration, temperature, mixing rate, and incubation period, the experiment was scaled up from shake flasks to fermenter. The optimum of previous experiments was applied, that is, pH 5, 25% initial molasses concentration, 35°C, and 116 rpm. But longer incubation period of 72 h was undertaken to study the fermentation trend at longer time span (>60 h). In fermenter, it is easier to control various parameters, like the temperature and pH. It was found that the pH values remained within the optimal range for HSC-22 yeast cells activities during the total fermentation time (i.e., pH 4.5–5); so, there was no requirement for the correction of this parameter during the fermentation process. Similar observation was reported by Cazetta et al. [19] and attributed this to the molasses chemical composition itself, making molasses exhibiting a buffering effect. This regulatory action depends on the main stabilizer compounds of the pH, which are the weak acids, and amino acids that would act in the acid range between pH 3 and 5, or the phosphates, whose buffering effects occur in the range of pH 6 and 7. It is obvious from data illustrated in Figure 5 that ethanol production increased with the depletion of sucrose, maltose, xylose (Figure 5(a)), and total sugars TS (Figure 5(b)). But the total reducing sugars TRS (Figure 5(b)), glucose, and fructose (Figure 5(a)) concentrations did not follow a certain trend. It has been reported that yeast can ferment sucrose throughout its assimilation, which can be degraded by invertase enzymes to be taken up as glucose and fructose, and these monosaccharides are the direct substrates utilized by yeast for fermentation [7, 17]. That might explain the unusual trend of increase and decrease in the glucose and fructose concentrations during the time course of the fermentation process. The rapid sucrose hydrolysis was accompanied by transient increment in the fructose and glucose concentrations (Figure 5(a)). That might indicate that the rate of sucrose hydrolysis exceeded the rates of consumption of the monosaccharides by yeast cells. The rate of hydrolysis of nonreducing disaccharide (sucrose; 48.7 mg L^−1 ^h^−1^) was higher than that of reducing disaccharide (maltose; 12.8 mg L^−1 ^h^−1^). But there was an overall decrease in different types of sugars in molasses feed stock with maximum ethanol yield of 44%, that is, conversion yield of *Y*_ethanol/TS_ ≈ 0.44 g/g and fermentation efficiency of 68.75%, within 60 h. A relative decrease of ethanol yield with longer incubation period was also observed (Figure 5(b)), recording 40.87% (*Y*_ethanol/TS_ ≈ 0.41 g/g) and fermentation efficiency of 63.86%, at the end of the fermentation process 72 h. This low ethanol yield relevant to the consumption of TS (75%) might be due to the inhibitory effect of other byproducts. Ergun and Mutlu [10] reported that fermentation inhibition might occur by secondary fermentation products, which would consequently limit the ethanol productivity. But the bioethanol yield recorded in this study is within the reported yield in the literature; Roukas [21] reported ethanol yield *Y*_ethanol/TS_ ≈ 0.3 g/g from beet molasses by* Saccharomyces cerevisiae*. Kopsahelis et al. [22] reported ethanol yield *Y*_ethanol/TS_ ≈ 0.47 g/g from waste molasses by* Saccharomyces cerevisiae*.

## 4. Conclusion

The central composite design CCD of experiments was found to be applicable for modeling the productivity of ethanol throughout the batch fermentation of sugarcane molasses SCM by the Egyptian yeast isolate* Pichia veronae* strain HSC-22 (accession number KP012558). By the use of the developed quadratic model, response surfaces, and contour plots, the investigation for the variation of the bioethanol yield depending on the changes in the process variables, pH, molasses concentration, temperature, mixing rate, and time, and the maximization of the bioethanol yield was found to be easily studied and predicted. Further work is undertaken now in EPRI Biotechnology Lab to investigate the effect of the molasses constituents and the secondary fermentation byproducts on the fermentation process and the bioethanol yield. Also, modeling and simulation for the rate of ethanol production with the rate of the sugars consumption and yeast growth are under investigation and will be published soon.

## Figures and Tables

**Table 1:** Parameters and levels of the experimental design.

Parameters	Levels		
	−1	0	+1
pH	4	5	6
Molasses concentration, wt.% (w : v)	15	20	25
Temp., °C	25	30	35
Mixing rate, rpm	50	100	150
Incubation period, h	24	48	72

**Table 2:** Experimental design matrix with experimental and predicted bioethanol yield.

Run number	Initial pH *A*	Molasses concentration *B*	Incubation temperature *C*	Mixing rate *D*	Incubation period *E*	Bioethanol concentration g/L	Bioethanol yield	
							%	
							Actual	Predicted
1	0	−1	0	0	0	18.2	41.4	39.1
2	−1	−1	−1	−1	+1	4.01	9.13	11.2
3	1	−1	−1	−1	+1	6.29	14.3	13.2
4	0	0	0	−1	0	19.3	33.0	34.1
5	0	0	0	0	+1	18.4	31.4	29.7
6	−1	0	0	0	0	15.3	26.1	22.6
7	0	0	−1	0	0	20.3	34.7	34.8
8	+1	−1	−1	+1	−1	4.61	10.5	13.9
9	+1	+1	+1	+1	+1	22.3	30.5	26.5
10	−1	+1	+1	−1	+1	19.5	26.7	23.9
11	−1	−1	+1	−1	−1	3.96	9.02	10.9
12	−1	−1	+1	−1	+1	6.48	14.8	16.5
13	+1	−1	+1	−1	−1	4.64	10.6	10.3
14	0	+1	0	0	0	23.5	32.1	38.9
15	+1	−1	+1	+1	+1	8.90	20.3	24.2
16	0	0	0	0	0	20.6	35.2	35.4
17	0	0	0	+1	0	22.1	37.7	37.7
18	−1	−1	−1	+1	−1	4.31	9.82	10.2
19	0	0	0	0	0	21.3	36.4	35.4
20	−1	+1	+1	+1	−1	8.80	12.0	13.5
21	0	0	+1	0	0	21.5	36.7	37.7
22	+1	+1	+1	+1	−1	9.44	12.9	13.5
23	+1	−1	+1	−1	+1	7.96	18.1	15.9
24	0	0	0	0	0	21.4	36.6	35.4
25	+1	0	0	0	0	11.1	19.0	23.6
26	0	0	0	0	0	20.9	35.6	35.4
27	0	0	0	0	0	21.3	36.4	35.4
28	+1	+1	−1	+1	−1	11.9	16.2	13.0
29	−1	+1	+1	+1	+1	19.4	26.5	26.6
30	0	0	0	0	0	21.3	36.4	35.4
31	−1	+1	−1	−1	−1	8.45	11.6	11.4
32	+1	+1	+1	−1	+1	14.7	20.1	22.2
33	+1	+1	−1	−1	−1	10.8	14.8	12.5
34	−1	−1	−1	−1	−1	3.39	7.72	7.13
35	+1	+1	−1	+1	+1	17.1	23.4	24.5
36	0	0	0	0	0	20.9	35.7	35.4
37	+1	−1	−1	−1	−1	3.60	8.20	9.26
38	+1	−1	−1	+1	+1	10.7	24.4	22.9
39	0	0	0	0	−1	10.8	18.4	21.1
40	0	0	0	0	0	20.6	35.3	35.4
41	+1	+1	+1	−1	−1	10.6	14.5	14.2
42	−1	−1	+1	+1	+1	9.54	21.7	23.4
43	+1	−1	+1	+1	−1	7.07	16.1	13.7
44	+1	+1	−1	−1	+1	13.6	18.5	19.0
45	0	−1	+1	+1	−1	6.26	14.3	12.7
46	0	+1	−1	−1	+1	12.9	17.7	17.9
47	0	+1	−1	+1	−1	7.31	9.99	10.3
48	0	+1	−1	+1	+1	15.7	21.5	21.9
49	0	+1	+1	−1	−1	12.2	16.7	15.8
50	0	−1	−1	+1	+1	8.74	19.9	19.3

**Table 3:** Analysis of variance of fitted quadratic regression model equation (4).

Source	SS^*∗*^	df^*∗*^	MS^*∗*^	*F*-value	*P* value	Remarks
Model	4.79*E* + 003	20	240	29.1	<0.0001	Very highly significant
*A*	8.80	1	8.80	1.07	0.309	Nonsignificant
*B*	90.4	1	90.4	11.0	0.00245	Significant
*C*	71.2	1	71.2	8.67	0.00632	Significant
*D*	115	1	115	13.9	0.000818	Highly significant
*E*	624	1	624	75.9	<0.0001	Very highly significant
*A* ^2^	376	1	376	45.8	<0.0001	Very highly significant
*B* ^2^	8.60	1	8.60	1.05	0.315	Nonsignificant
*C* ^2^	1.81	1	1.81	0.220	0.643	Nonsignificant
*D* ^2^	0.569	1	0.569	0.0693	0.794	Nonsignificant
*E* ^2^	246	1	246	30.0	<0.0001	Very highly significant
*AB*	1.91	1	1.91	0.233	0.633	Nonsignificant
*AC*	14.6	1	14.6	1.78	0.193	Nonsignificant
*AD*	4.98	1	4.98	0.606	0.443	Nonsignificant
*AE*	0.0276	1	0.0276	0.00336	0.954	Nonsignificant
*BC*	0.878	1	0.878	0.107	0.746	Nonsignificant
*BD*	33.3	1	33.3	4.06	0.0534	Possibly significant
*BE*	12.2	1	12.2	1.49	0.232	Nonsignificant
*CD*	3.04	1	3.04	0.370	0.548	Nonsignificant
*CE*	4.91	1	4.91	0.598	0.446	Nonsignificant
*DE*	50.3	1	50.3	6.12	0.0195	Possibly significant
Residual	238	29	8.22			
Pure error	2.13	7	0.304			
Corrected total	5.03*E* + 003	49				

^*∗*^SS: sum of squares; df: degree of freedom; MS: mean square.
